# GPS-based street-view greenspace exposure and wearable assessed physical activity in a prospective cohort of US women

**DOI:** 10.1186/s12966-025-01795-8

**Published:** 2025-07-06

**Authors:** Li Yi, Jaime E. Hart, Grete Wilt, Cindy R. Hu, Marcia Pescador Jimenez, Pi-I Debby Lin, Esra Suel, Perry Hystad, Steve Hankey, Wenwen Zhang, Jorge E. Chavarro, Francine Laden, Peter James

**Affiliations:** 1 Division of Chronic Disease Research Across the Lifecourse (CoRAL), Department of Population Medicine, Harvard Medical School and Harvard Pilgrim Health Care Institute, 401 Park Drive 4th Floor East, Boston, MA 02215 USA; 2 Department of Nutrition, Harvard T. H. Chan School of Public Health, Boston, MA USA; 3 Channing Division of Network Medicine, Department of Medicine, Brigham and Women’s Hospital and Harvard Medical School, Boston, MA USA; 4 Department of Environmental Health, Harvard T. H. Chan School of Public Health, Boston, MA USA; 5 Department of Epidemiology, Boston University School of Public of Health, Boston, MA USA; 6 Centre for Advanced Spatial Analysis, University College London, London, UK; 7 College of Health, Oregon State University, Corvallis, OR USA; 8 School of Public and International Affairs, Virginia Polytechnic Institute and State University, Blacksburg, USA; 9 Edward J. School of Planning and Public Policy, Rutgers, The State University of New Jersey, New Brunswick, NJ USA; 10 Department of Public Health Sciences, University of California Davis School of Medicine, Davis, CA USA

**Keywords:** Greenspace, Nature, Physical activity, Wearables, Mobile health, Street view image, Machine learning, Deep learning, Global positioning system

## Abstract

**Background:**

Increasing evidence positively links greenspace and physical activity (PA). However, most studies use measures of greenspace, such as satellite-based vegetation indices around the residence, which fail to capture ground-level views and day-to-day dynamic exposures, potentially misclassifying greenspace and limiting policy relevance.

**Methods:**

We analyzed data from the US-based Nurses’ Health Study 3 Mobile Health Substudy (2018–2020). Participants wore Fitbits™ and provided smartphone global positioning system (GPS) for four 7-day periods throughout the year. Street-view greenspace (%trees, %grass, %other greenspace [flowers/plants/fields]) were derived from 2019 street-view imagery using deep-learning algorithms at a 100-meter resolution and linked to 10-minute GPS observations. Average steps-per-minute for were calculated for each 10-minute period following each GPS observation. Generalized Additive Mixed Models examined associations of street-view greenspace exposure with PA, adjusting for individual and area-level covariates. We considered effect modification by region, season, neighborhood walkability and socioeconomic status (SES), temperature, and precipitation.

**Results:**

Our sample included 335 participants (mean_age_= 39.4 years, *n* = 304,394 observations). Mean steps-per-minute per 10-minutes were 6.9 (SD = 14.6). An IQR increase (18.7%) in street-view trees was associated with a 0.36 steps-per-minute decrease (95%CI: -0.71, -0.01). In addition, an IQR increase (10.6%) in grass exposure was associated with a 0.59 steps-per-minute decrease (95% CI: -0.79, -0.40); however, the association was non-linear and flattened out after the 75th percentile of street-view grass. Conversely, an IQR increase (1.2%) in other greenspace was associated with a 1.99 steps-per-minute increase (95%CI: 0.01, 3.97). Associations were stronger in the spring and in higher SES neighborhoods, and among residents of the Northeast.

**Conclusions:**

In this prospective cohort, momentary street-view exposure to trees and grass was inversely associated with PA, while exposure to other greenspace was positively associated. Future research should confirm these results in other populations and explore the mechanisms through which specific greenspace components influence PA.

**Supplementary Information:**

The online version contains supplementary material available at 10.1186/s12966-025-01795-8.

## Background

Physical inactivity is a major risk factor for many chronic diseases [1], yet over 50% of US adults fail to meet the recommended PA levels [2]. Greenspace has been linked to PA promotion by providing safe, aesthetically pleasing environments for recreation, stress reduction, and fostering social interactions that encourage PA behavior [3–6]. However, studies on the relationship between greenspace and PA have yielded inconsistent results [7], likely due to the reliance of satellite-derived composite measure, such as Normalized Difference Vegetation Indices surrounding participants’ home address(NDVI) [8]. While NDVI, ranging from − 1 to 1 with higher positive values reflecting environments with dense, green vegetation, is effective for evaluating overall greenness, it does not specifically capture ground-level exposure, which may better represent an individual’s interaction with their environment [8].

Recently, emerging research has leveraged ubiquitous street-view imagery combined with advances in deep learning models to assess ground-level greenspace exposure with two focused on their impact on PA [9, 10]. However, these studies used exposures around the home to derive participants’ greenspace exposure [11]. This method may introduce measurement error by not accounting for exposure beyond residential areas due to daily mobility (visiting various locations and trips), thus impacting the accurate quantification of greenspace and PA associations [11–14]. Also, both studies aggregated multiple greenspace types as one metric (e.g., percentages of greenspace in view) rather than distinguishing between greenspace types, which may influence PA differently (e.g., trees provide shade and reduce stress, whereas grass often indicates parks and open spaces) [15]. As a result, they may be limited in informing targeted interventions to optimize greenspace design to promote PA across diverse populations and settings.

In this study, we applied pre-trained deep learning segmentation models to street-view images across the US and linked them to momentary smartphone GPS location data to assess associations with objective, momentary consumer wearable PA data from the Nurses’ Health Study 3 (NHS3) Mobile Health (mHealth) Substudy. This intra-individual, intensive longitudinal study aimed to quantify associations between momentary smartphone GPS-based exposures to specific types of greenspace (e.g., trees, grass, and flowers) visible in street-view images and time-matched PA measured by wearable devices. Based on the finding of a previous residential-based study on greenspace and PA [16], we hypothesized that increased GPS-based momentary exposure to street-view trees would be associated with higher PA over 10-minute periods, accounting for individual- and areal-level potential confounders. Furthermore, as relationships between environmental exposures and PA outcomes may vary by season and geographical region [17, 18], and environmental co-exposures, including temperature and humidity [19, 20], neighborhood socioeconomic status (SES) [21, 22], and walkability [21, 23], can influence greenspace-PA associations, we examined these factors as potential effect modifiers.

## Methods

### Study population

#### Nurses’ health study 3 (NHS3)

The NHS3, initiated in 2010, is an ongoing open-enrollment cohort of nurses and nursing students in the US or Canada. Eligible participants must be registered nurses, licensed practical/vocational nurses, or nursing students born on or after January 1, 1965. In 2018, at the time potential Substudy participants were identified, NHS3 had enrolled 49,693 participants. Enrolled individuals updated their residential history and completed biannual web-based questionnaires on lifestyle and medical characteristics. The response rate for participants who completed two or more questionnaires exceeded 80% [24].

#### NHS3 mobile health (mHealth) substudy

The NHS3 mHealth Substudy, conducted between 2018 and 2020, enrolled 513 participants from 42 contiguous US states. Eligibility criteria included age ≥ 21 years as of March 2018 and prior completion of NHS3 questionnaires on height, weight, PA, and sleep [25]. Individuals diagnosed with sleep disorders were excluded because of the reduced accuracy of the Fitbit™ wearable devices in such populations.

The recruitment, protocol, and data collection methods of the study are detailed in a related protocol paper [25]. Briefly, participants wore Fitbit™ devices and downloaded a custom smartphone app, enabling data collection during seven-day sampling periods every three months for one year to capture seasonal behavior and exposure variations. The 7-day protocol, consistent with other mobility studies [11], collected data from both workdays and non-workdays.

Among the 513 participants enrolled, 464 provided high-quality GPS data (*n* = 944,476 observations). For the primary analytical sample, following a previous study [26], we excluded 127 participants who did not provide at least eight hours of GPS data on at least three unique days and omitted main sleep periods (*n* = 331,223 observations) assumed to be free of PA based on Fitbit™ sleep records. We also excluded observations without Fitbit™ step-count or street-view greenspace exposure (*n* = 328,421 observations) data. The final analytical sample included 335 participants, with 304,394 observations (see Appendix 1 for the detailed participant flow diagram). Demographics of the analytical sample compared with those excluded are shown in Appendix 2. In general, the included participants were similar in age, less likely to be white (92.0% vs. 94.8%), more likely to be married (60.1% vs. 54.5%) and slightly more likely to be employed (97.1% vs. 94.6%) and have a higher degree (25.9% vs. 23.0%).

### Street-view greenspace exposure assessment

Our methods for deriving street-view greenspace metrics have been detailed in previous studies [27, 28]. Street-view greenspace metrics were derived annually using the Pyramid Scene Parsing Network (PSPNet) [29], a pre-trained deep learning model based on the ADE20K dataset [29], to segment images captured every 100 m along the street network in all US Core-Based Statistical Areas from 2007 to 2020. Each image was segmented at the pixel-level into 150 categories (e.g., trees, grass, plant, fields, flowers, and sidewalks). For each year, we created a 100-meter resolution raster, displaying the percentage of each category visible at each location. Missing images for specific years or locations were filled using carry-forward and backward methods. We also apply focal statistics-based smoothing to create two additional 500 m and 1,000 m raster.

grids by summarizing the mean values of 100 m raster with the respective distance window around each pixel.

In this study, 2019 street-view greenspace metrics were linked to GPS location data collected between late 2018 and early 2020, generating GPS-based greenspace exposure values. Exposure metrics were assessed at 100-meter resolution as the primary exposure, with 500-meter and 1,000-meter resolution used in sensitivity analyses. Three greenspace features were analyzed: (1) trees, (2) grass, and (3) other greenspace (plants, flowers, and fields), as these features have been associated with physical activity outcomes in prior studies [30, 31].

### Physical activity (PA) outcome

Physical activity (PA) data were collected using Fitbit™ devices (Fitbit Charge HR, Charge 2, and Charge 3). These devices measure PA through accelerometry and use proprietary algorithms to calculate minute-level step counts, activity intensity, and sleep duration, and have been shown to be reasonably accurate in assessing PA [32]. Step counts were averaged over 10-minute intervals following each GPS location. This averaging reduces fluctuations caused by gaps in the GPS data and ensures stable and reliable activity measurements [33]. The primary PA metric was the mean steps-per-minute within each 10-minute interval.

### Covariates

Individual- and areal-level covariates were selected a priori based on their potential to be common causes of greenspace exposure and PA. Individual-level confounders included age (continuous, in years), socioeconomic status (SES) as indicated by education level (binary: master’s degree in nursing or higher), and marital status (binary: never married vs. ever married, widowed, or divorced). We obtained data from the initial NHS3 cohort questionnaire, prior to Substudy enrollment [24].

Area-level confounders included neighborhood SES, walkability, mean daily temperature, daily precipitation, season, and Census region. To estimate neighborhood SES, we generated a composite score from seven 2010 Census tract-level variables linked to health outcomes: education, employment, housing, wealth, racial composition, and population density [34]. Each variable was standardized into z-scores and summed to create a neighborhood SES score, where higher values indicated less deprivation. The neighborhood SES scores were linked to each 10-minute GPS point for GPS-based neighborhood SES. For neighborhood walkability, we created a Census tract level composite score from intersection density based on 2019 Tiger/Line shapefiles of all roads with interstates removed [35], population density from 2019 American Community Survey population data [36], and business density from 2018 Infogroup US Historical Business Data [37]. Variables were z-scored and summed to form a walkability index [38], with higher scores indicating greater walkability. Walkability scores were assigned to each 10-minute GPS point. We obtained daily mean temperature and precipitation data at 800 m resolution for 2018–2020 from the PRISM climate dataset [39], linked by date and GPS coordinates to each 10-minute measurement. Precipitation was dichotomized as any or none. Census region (Northeast, Midwest, South, West) and season (Spring [March-May], Summer [June-August], Fall [September – November], Winter[December – February]) were assigned based on the date and location of each GPS point.

### Statistical analyses

We applied Generalized Additive Mixed Models (GAMM) to examine potential linear and non-linear associations between GPS-based street-view greenspace exposure and PA. We included a random intercept for each participant to account for repeated measurements within the same participant. We first modeled street-view greenspace (%trees, %grass, %other greenspace [flowers/plants/fields]) in the same model simultaneously using penalized cubic splines to capture non-linear relationships, utilizing the *mgcv* package (version 1.9-1) in R. Preliminary models revealed a non-linear association between street-view grass and PA but not for trees or other greenspace. Consequently, we retained a non-linear term for street-view grass, but modeled trees and other greenspace as linear. All models were adjusted for individual- and area-level variables listed above. An autoregressive correlation structure was specified to address temporal correlations within-participant due to the repeated-measures design.

#### Effect modification

We examined effect modification by stratifying models by neighborhood walkability quartiles, street-view sidewalks, neighborhood SES score quartiles, mean daily temperature quartiles, daily precipitation status, region, and season. We assessed the statistical significance of effect modification by including multiplicative interaction terms between greenspace metrics and modifiers. Interaction significance (P-interaction < 0.05) was determined using Chi-Square tests to compare models with and without interaction terms.

#### Sensitivity analyses

To address potential biases, we conducted sensitivity analyses under four scenarios to test the robustness of our findings. The first analysis aimed to minimize selective daily mobility bias [40], which refers to the difficulty in separating passive exposure from active seeking of a space. To mitigate this, we limited each participant’s activity space to GPS locations within a standard deviation ellipse, representing the standard deviation of x- and y-coordinates from the mean center of their GPS points. This method helps to exclude locations outside the typical range of movement and reduces selective daily mobility bias in our analysis. For the second sensitivity analysis, we examined associations during non-work hours by excluding time spent at work. We geocoded workplace addresses and limited GPS data to areas outside a 160-meter buffer (0.1 mile) around these locations. The buffer size was based on typical hospital dimensions [41], the main workplace for most participants. In the third sensitivity analysis, we limited data to time in active transportation or recreation. To do this, we only included GPS datapoints with velocities between 0.8 and 4 m/s, which correspond to walking and running, reducing the inclusion of sedentary activities or driving time [42]. In our fourth sensitivity analysis, following a previous study [26], we limited the cohort to the 208 participants who provided a minimum of 12 h of GPS data daily across five unique days in two sampling periods. This stricter criterion ensured substantial data for each individual, enhancing the robustness and reliability of the intra-individual comparisons in our primary analysis.

In addition to addressing biases, we also performed analyses adjusting for satellite-based greenness (NDVI) and street-view sidewalk in models to examine the additional information contributed by street-view greenspace metrics independent of satellite-based measures or walking infrastructure. NDVI was derived from Landsat NDVI data estimated as the annual average NDVI in a 270 m buffer around each residential address [26]. The street-view sidewalk metric was derived using the same algorithm described above for the street-view greenspace metrics. Finally, we repeated our analyses using GPS-based street-view greenspace metrics at 500-meter and 1,000-meter buffers to examine the robustness of results to buffer size choices [43]. All statistical analyses were performed using R version 4.4.1.

## Results

### Descriptive statistics

We included over 304,394 10-minute observations in our analyses (*N* = 335 participants), on average participants took 6.9 (SD 14.6) steps-per-minute. Participants were on average 39.4 (7.0) years old, predominantly White (92%), and mostly employed (97%), over 60% were married and 26% held advanced degrees (Table 1). Participants in the highest quartile of momentary exposures to street-view trees, grass, and greenspace were more likely to hold advanced degrees and were typically found in neighborhoods with higher SES and lower walkability. When restricting the dataset to the 206 participants who provided at least 12 h of GPS data daily across five unique days in two sampling periods (*n* = 232,403 observations), participant characteristics remained consistent (Appendix 2).

**Table 1:** Study demographics for the nurses’ health study 3 mHealth substudy (overall and by lowest versus highest quartiles of three street-view greenspace metrics)

	(*N* = 335; 304,394 Observations)	Street-view Trees Q1 (0.0-6.5%)	Street-view Trees Q4 (25.1–76.6%)	Street-view Grass Q1 (0.0-1.7%)	Street-view Grass Q4 (12.4–32.9%)	Street-view Other Green space Q1 (0.0-0.2%)	Street-view Other Green space Q4 (1.4–39.5%)
Age, mean (SD)	39.4 (7.0)	39.5 (6.9)	39.1 (7.2)	39.0 (6.9)	39.9 (7.7)	39.8 (7.5)	40.6 (7.0)
Race, (%)							
White	92.0	93.0	91.9	89.3	94.3	92.3	92.2
Black	3.2	5.1	0.3	4.2	2.6	4.6	0.8
Asian	0.7	0.2	0.7	0.2	0.7	0.4	2.2
Mixed Race	0.9	0.8	1.9	0.7	0.6	1.8	0.2
Other	3.2	0.9	5.2	5.6	1.8	1.0	4.5
Hispanic, (%)	5.7	4.0	5.5	5.1	5.4	5.0	2.7
Married, (%)	60.1	54.4	63.1	61.2	60.9	58.1	70.9
Advanced Degree, (%)	25.9	22.8	27.8	21.8	24.0	27.2	30.5
Employment, (%)	97.1	95.2	97.1	95.1	97.7	98.5	95.8
Walkability z-score, mean (SD)	0.7 (3.7)	1.6 (4.9)	0.3 (2.8)	3.2 (6.5)	-0.6 (1.0)	0.8 (4.4)	0.6 (3.5)
Neighborhood SES z-score, mean (SD)	1.9 (3.3)	0.7 (3.1)	2.8 (3.4)	1.6 (3.7)	2.4 (3.1)	1.6 (3.4)	2.4 (3.3)
Temperature (°C), mean (SD)	14.8 (9.9)	15.0 (10.4)	14.0 (9.7)	15.1 (9.7)	14.9 (10.3)	14.8 (10.5)	14.3 (9.3)
Precipitation (mm), mean (SD)	3.4 (9.0)	3.1 (9.2)	3.4 (8.8)	2.5 (6.9)	4.1 (10.4)	3.1 (8.7)	3.1 (7.9)
NDVI (0–1), mean (SD)	0.3 (0.2)	0.2 (0.2)	0.4 (0.2)	0.2 (0.2)	0.4 (0.2)	0.3 (0.2)	0.4 (0.2)
Seasonality, (%)							
Fall	25.9	26.6	24.8	25.7	24.3	25.0	26.5
Spring	22.0	21.2	23.9	22.2	23.3	24.0	22.8
Summer	32.8	32.0	30.1	31.8	33.5	31.4	30.5
Winter	19.3	20.2	21.2	20.3	18.9	19.6	20.2
Mean steps per minute, mean(SD)	6.9 (14.6)	7.1 (14.8)	6.7 (15.2)	7.3 (15.1)	6.3 (13.7)	6.9 (14.4)	7.1 (15.4)

Abbreviations: SES, socioeconomic status; NDVI, Normalized Difference Vegetation Index

Each participant contributed an average of 53.6 observations per day (SD 44.1; median 47.0) or approximately 8.9 h/day (SD 7.4; median 7.8). Over the one-year study period, participants contributed an average of 908.6 observations (SD 721.8; median 713.0) or approximately 151.4 h (SD 120.3; median 118.8). Observations were highest during the summer months (32.8%) and lowest during the winter months (19.3%; Appendix 2).

PA outcomes varied by season and region, with participants in the Northeast and summer months demonstrating the highest mean steps-per-minute (Appendix 3). Street-view greenspace metrics also differed by season and region, with participants residing in the Midwest exhibiting the highest average exposure to street-view trees and grass across all observations (Appendix 3).

Correlations between the three street-view metrics and other spatial factors are shown in Appendix 4. Street-view trees and grass were positively and moderately correlated with NDVI (*r* = 0.44 for trees and *r* = 0.45 for grass), suggesting that they measured similar but not completely overlapping aspects of greenspace. Street-view grass was negatively and moderately correlated with walkability (*r*=-0.37). Street-view greenspace metrics and other spatial factors (e.g., neighborhood SES) were weakly correlated (*r* < 0.30; see Appendix 4).

### Street-view greenspace metrics and PA

**Fig. 1:**
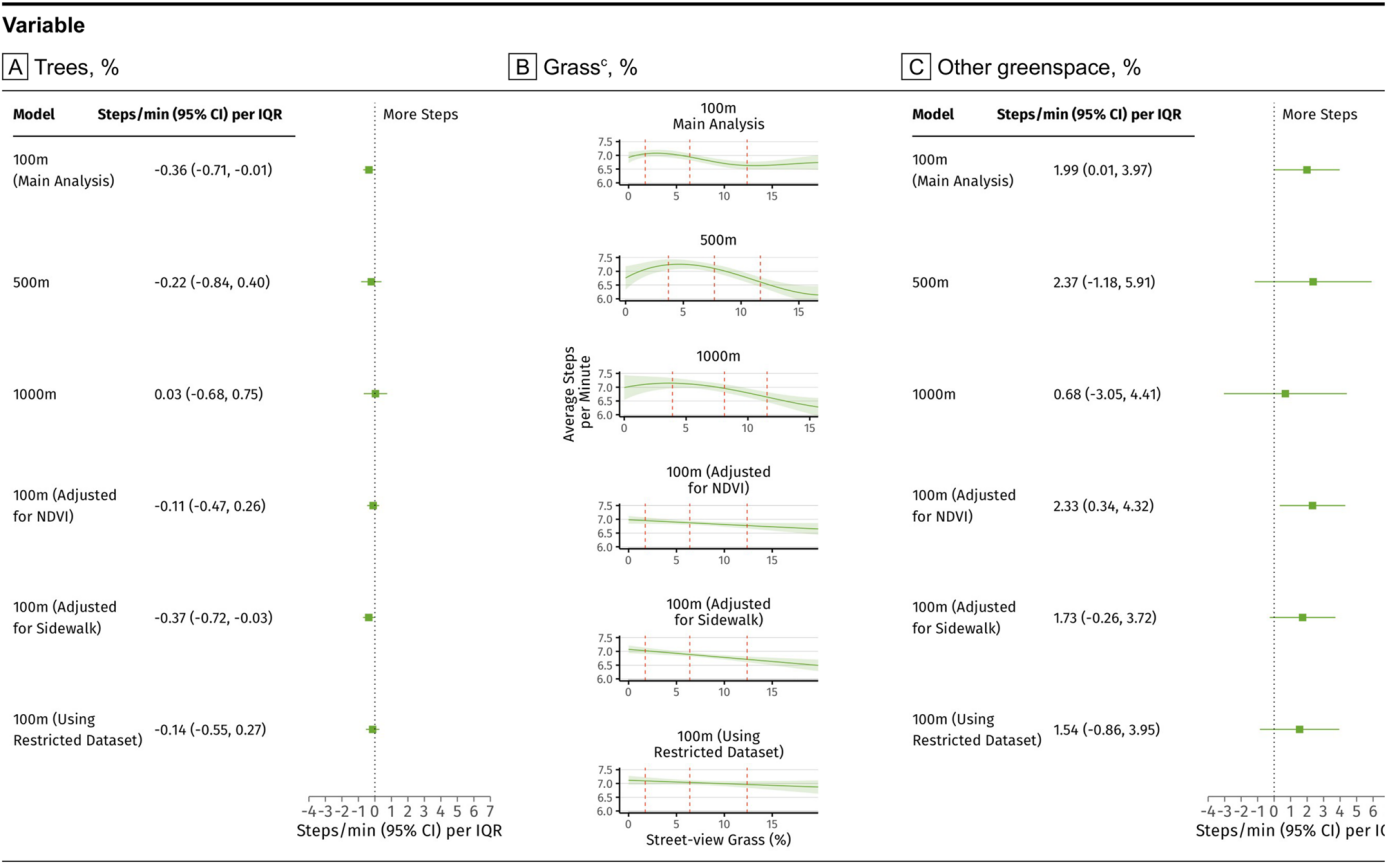
Results of main and sensitivity analyses^a^ on associations between three street-view greenspace metrics^b^ and average steps-per-minute across a 10-min period^d^ in the Nurses’ Health Study 3 mHealth Substudy (*N* = 335, *n* = 304,394 10-minute observations). Abbreviations: SES, socioeconomic status; NDVI, Normalized Difference Vegetation Index. (**a**) Model notes. 500 m: Models using 500 m street-view metrics. 1000 m: Models using 1000 m street-view metrics. 100 m (Adjusted for NDVI): Main analysis model additionally adjusted for Normalized Difference Vegetation Index (NDVI). 100 m (Adjusted for Sidewalk): Main analysis model additionally adjusted for % street-view sidewalk. 100 m (Using restricted dataset): Main analysis model using restricted analytical dataset (participants who provided at least 12 h of GPS location data daily on 5 unique days in two distinct sample periods). (**b**) Generalized Additive Mixed Models (GAMM) was applied to examine potential linear and non-linear associations between GPS-based street-view greenspace exposure and PA. We included a random intercept for each participant to account for repeated measurements within the same participant. Three street-view greenspace metrics were mutually adjusted in all models. Trees and other greenspace were modeled with linear terms; while grass was modeled with a non-linear term. (**c**) 25th, 50th, and 75th percentiles of the street-view grass metric were displayed as red dash lines. The x-axis was limited to 5th and 95th percentile of street-view grass metric to restrict influences of extreme estimates on visual. (**d**) Models controlled for age, education level, marital status, and area-level measures of neighborhood socioeconomic status, walkability, mean daily temperature, daily precipitation, season and Census region in the 2018–2020 Nurses’ Health Study mHealth Substudy. (**e**) IQR for street-view trees (18.7%), street-view grass (10.6%), and street-view othergreen (1.2%)

Momentary exposure to street-view trees was negatively associated with mean steps-per-minute, with an IQR increase associated with a 0.36 steps-per-minute decrease (95% CI: -0.71, -0.01; Fig. 1A). Moreover, we observed a statistically significant non-linear association between GPS-based momentary exposure to street-view grass and mean steps-per-minute (Fig. 1B). Specifically, we observed momentary street-view grass exposure was associated with lower PA up to value of 10.0% (65th percentile), with an IQR increase in street-view grass associated with a decrease of 0.59 steps-per-minute (95% CI: -0.79, -0.40). Above 10%, no association was observed between momentary street-view grass exposure and PA. Furthermore, momentary exposure to street-view other greenspace showed a positive linear association with mean steps-per-minute, with an IQR increase associated with a 1.99 steps-per-minute increase (95% CI: 0.01, 3.97; see Fig. 1C).

### Stratified analyses

**Fig. 2:**
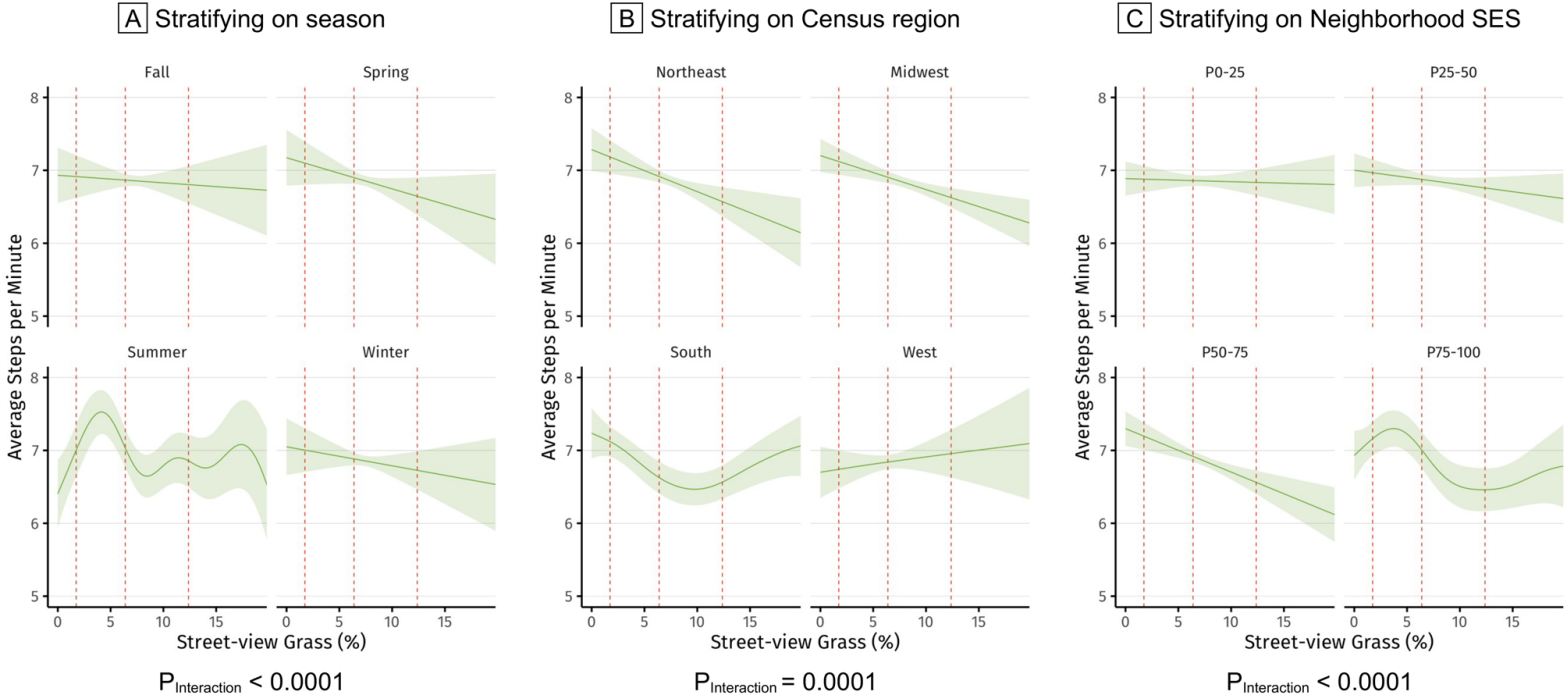
Associations^a^ between street-view grass and average steps-per-minute across a 10-min period^b^ stratifying on season, Census region, and neighborhood socioeconomic status (SES)^c^ in the Nurses’ Health Study 3 mHealth Substudy (*N* = 335, *n* = 304,394 10-minute observations). (**a**) Generalized Additive Mixed Models (GAMM) was applied to examine potential linear and non-linear associations between GPS-based street-view greenspace exposure and PA. We included a random intercept for each participant to account for repeated measurements within the same participant. Three street-view greenspace metrics were mutually adjusted in all models. Trees and other greenspace were modeled with linear terms; while grass was modeled with a non-linear term. (**b**) Models controlled for age, education level, marital status, and area-level measures of neighborhood socioeconomic status, walkability, mean daily temperature, daily precipitation, season and Census region in the 2018–2020 Nurses’ Health Study mHealth Substudy. (**c**) Red dashlines representing 25th, 50th and 75th percentiles of street-view grass metric

Effect modification by season, region, and neighborhood SES was observed for the association between momentary street-view grass exposure and mean steps-per-minute (Fig. 2). The inverse association was strongest during spring, where an IQR increase in momentary street-view grass was associated with a decrease of 0.45 steps-per-minute (95% CI: -0.85 to -0.06; P_Interaction_<0.0001; see Fig. 2A). The association was most pronounced in the Northeast compared to the other regions, with an IQR increase in momentary street-view grass linked to a 0.61 decrease in steps-per-minute (95% CI: -0.92 to -0.31; P_Interaction_=0.0001). Furthermore, the inverse association was only observed in neighborhoods with higher SES (3rd and 4th quartiles; P_Interaction_<0.0001), whereas no statistically significant association was detected in lower-SES neighborhoods. No seasonal or regional differences were observed in the associations of momentary exposure to street-view trees or other greenspace with PA, nor was there evidence of effect modification by neighborhood walkability, % street-view sidewalks, mean daily temperature, or daily precipitation for any greenspace metric.

### Sensitivity analyses

The non-linear association between momentary street-view grass exposure and mean steps-per-minute, particularly the inverse association within the IQR of grass values, was robust across multiple sensitivity analyses (Fig. 1B). These included restricting the cohort to participants with more complete data, exploring 500-meter and 1,000-meter greenspace buffers, and adjusting for NDVI or street-view sidewalk exposure. Similarly, the linear negative association between momentary exposure to street-view trees and mean steps-per-minute (Fig. 1A), as well as the linear positive association between momentary exposure to street-view trees and mean steps-per-minute, remained consistent across these analyses (Fig. 1C). Sensitivity analyses addressing potential biases further supported the robustness of our findings (Appendix 5). Models accounting for selective daily mobility bias confirmed an inverse association for momentary exposure to street-view grass and a positive association for other greenspace. Restricting the GPS datapoints to active transport velocities (walking and running) yielded similar results. However, associations for momentary exposure to street-view other greenspace were attenuated when GPS datapoints near work locations were excluded.

## Discussion

In this intensive longitudinal analysis of observational data from a mobile health substudy embedded in a US-based nationwide prospective cohort, we examined minute-level associations between street-view greenspace exposure and PA. Our findings suggest a moderately positive association between momentary exposure to other greenspace (plants, flowers, and fields) visible in street-view images and PA, as well as a small negative association between momentary exposure to visible trees and PA. We also observed a small non-linear inverse association between momentary exposure to grass visible in street-view images and PA. These associations generally remained robust across sensitivity analyses, including the use of larger buffer sizes, adjustment for satellite-based NDVI and sidewalks visible in street-view imagery, and restrictions to datasets accounting for potential biases. The non-linear inverse association for street-view grass was more pronounced in spring and neighborhoods with higher SES, and among participants residing in Northeast region.

The inverse association between street-view trees and grass exposure and PA, respectively, adds nuance to the existing large body of literature on residential greenness and PA, and the limited number of GPS-based greenspace and PA studies [21, 23, 26, 44], where findings have been inconsistent [45]. This inconsistency may be due to prior studies’ reliance on NDVI, which is an aggregate measure of greenness that cannot differentiate between greenspace components. Using street-view imagery, we isolated exposure to trees and grass separately and observed an overall inverse relationship for both, suggesting that these elements may not consistently promote PA.

In terms of trees, while they can provide shade, buffer noise, and improve air quality [16], their influence on PA may depend on contextual factors, such as the surrounding infrastructure (e.g., sidewalks) and neighborhood characteristics (neighborhood crime) [46]. Due to the occupational nature of our cohort (nurses and nursing professionals), participants may have accumulated most of their walking steps in the workplace where tree canopy coverage might have been limited or located in high-traffic areas that may be less conducive to PA [47]. This seems to be confirmed by the directional change in the association (from negative to positive) between momentary street trees and PA once we have excluded datapoints within work locations.

As for grass, visual inspection of street-view imagery with high grass values in our dataset indicated that these areas often included large open spaces, parks, or residential lawns. While such spaces could potentially promote active recreation, such as walking or jogging, they may be more likely to support sedentary activities, such as reading, sitting, or picnicking [48], or an indicator of a sprawling urban form that promotes auto-dependent lifestyles [49]. Additionally, areas with large volumes of grass may lack the infrastructure needed for walking, such as trails or paths, although results were similar in models where we adjusted for the presence of sidewalks [50]. Nevertheless, it is important to acknowledge that although grass may not be a primary driver of physical activity—or may even support more sedentary behaviors—it may still confer health benefits through mechanisms such as attention restoration and stress recovery, as well as by providing a setting for social interaction that fosters social cohesion [28, 51].

In contrast, momentary exposure to street-view other greenspace was positively associated with PA, mutually adjusting for street-view trees and grass. This category, which includes flowers, plants, and landscaped areas, may serve as an indicator of well-maintained neighborhoods, social cohesion, and aesthetic appeal [52]. Visual inspection suggested that street-view images with high values of other greenspace used in our study were often heavily landscaped, such as parks or sidewalks with decorative plants. These features may enhance the perceived quality of greenspace, which has been linked to higher PA levels in adults [53].

In our study, the associations between street-view grass and PA also differed by region, season, and neighborhood SES on. We found that the inverse association was strongest during spring, which suggests shifts in outdoor activity preferences or differences in greenspace use throughout the year. We speculate the spring season may have a higher proportion of social and sedentary activities, such as outdoor picnics or gatherings on grassy lawns, compared to summer or fall when active recreation may be more common. Lastly, we found that the non-linear inverse association between street-view grass and PA was strongest when participants were exposed to neighborhoods with higher SES. Higher SES neighborhoods often feature better aesthetics, lower crime rates, and more public spaces compared to those with lower SES [20], which makes grassy areas conducive to outdoor recreational activities that are predominantly sedentary in nature (e.g., outdoor dining or socializing).

Increasingly, there are concerns about the influence of selective daily mobility bias on GPS-based exposure and physical activity (PA) studies [40]. In our context, this bias arises when individuals inclined to be more active selectively visit environments conducive to exercise, which can inflate observed associations between environmental exposures and PA. To mitigate this bias, we restricted GPS data to locations within participants’ usual activity spaces, using the standard ellipse approach, and our results remained consistent. However, selective mobility bias may still have influenced our findings. Because we did not collect travel diaries or mobility surveys to determine trip purposes, an approach recommended by previous studies [54, 55] to address this bias, we could not distinguish whether participants’ exposures were incidental or self-selected for PA, limiting our ability to fully adjust for this confounding. Accordingly, our observed exposure–PA associations should be interpreted with caution.

Although the associations for street-view trees and grass exposure were relatively small, their cumulative impacts could be meaningful. For example, a decrease of 0.59 steps-per-minute within a 10-minute window per IQR increase in grass exposure translates to approximately 568 fewer steps per day for an individual with 16 waking hours. In contrast, a positive association of 1.99 steps-per-minute increase per IQR increase in other greenspace could result in a daily increase of 1,910 steps, a level associated with notable health benefits, including reduced cardiovascular risk [56]. These findings emphasize the importance of well-designed urban spaces for promoting PA [57].

### Strengths and limitations

Our study has several limitations. The use of step count as a proxy for PA does not capture other forms of PA, such as weightlifting, cycling, gardening, or swimming. However, walking remains the primary source of PA in most US adults, including NHS3 participants [2]. Relatedly, participants didn’t wear devices at all times; however, sensitivity analyses and comparisons of our Substudy to the full cohort in a previous study suggest that our analysis is representative [25]. Additionally, our street-view greenspace metrics were derived from annual snapshots, which may not fully capture seasonal variations in greenspace. However, a previous study applying this data reported most street-view images (∼78%) were collected during warmer months (April–October), which likely mitigated this limitation [27]. In addition, we assumed that the image captured at the time a Google Street View car drove by represented what an individual would see on the ground based on their GPS data on a given day, although streetscapes might vary daily. Furthermore, street view images only capture greenspace visible from the road, potentially missing parks and other greenspaces away from roads. And we were not able to account for slopes associated with street-view greenspace components in urban landscapes, which may affect physical activity [58]. Finally, the NHS3 cohort predominantly included upper-middle-class White women, limiting the generalizability of our findings to more diverse populations, as the role of street-view greenspace in promoting PA in lower income populations may be less effective due to other neighborhood characteristics (e.g., crime, poor infrastructure) and the distribution of exposures captured in our sample may not be representative of exposures in all areas of the US or around the world. Future studies should explore these associations in cohorts with greater racial, ethnic, and socioeconomic diversity to confirm and expand on our findings.

Our study has several strengths. First, the use of time-variant, GPS-based street-view greenspace metrics at 100-meter resolution allowed us to capture ground-level personalized exposure to specific types of greenspace with high spatial precision, offering a more relevant perspective than satellite-based metrics. Second, the integration of intensive longitudinal geolocation and PA data enabled us to quantify minute-level associations and examine modifying roles of temporally aligned contextual factors, such as walkability and temperature. Third, our use of objectively measured PA reduced the likelihood of recall bias compared with self-reported measures, which are commonly used in cohort studies. Finally, the nesting of the mHealth Substudy within the larger NHS3 cohort ensured high-quality data collection and robust covariate assessment.

## Conclusions

By linking minute-level street-view greenspace and PA data, we not only address critical gaps in the literature and look holistically at greenspace exposures beyond the residential environment but also investigate the specific types of greenspace that may drive PA. The positive association between momentary exposure to street-view other greenspace and PA underscores the potential value of investing in greenspaces that enhance both aesthetic and functional quality to increase the perceived attractiveness of greenspaces, encouraging higher PA levels. Conversely, our findings on the negative association between street-view trees and grass and PA indicate that certain greenspaces may be more relevant for sedentary recreational behavior than for active types. Thus, our results have important implications for urban planning and public health initiatives that focus on health-promoting greenspaces. We also suggest that future studies continue to probe the mechanisms through which different greenspace types may influence PA.

## Electronic supplementary material

Below is the link to the electronic supplementary material.

Supplementary Material 1

## Data Availability

Access to the Nurses’ Health Study 3, requires the proposal and approval of the specific research project and is not directly sharable. Details on obtaining access are available at https://nurseshealthstudy.org/researchers. Access to street-view derived metrics is available by contacting the study authors.

## Abbreviations

GPS: Global positioning system
NDVI: Normalized difference vegetation indices
NHS: Nurses’ health study
PA: Physical activity
