# Supplementary material for: GPS-based street-view greenspace exposure and wearable assessed physical activity in a prospective cohort of US women
Source: Int J Behav Nutr Phys Act. 2025 Jul 6;22:92. doi: 10.1186/s12966-025-01795-8 (PMC12232564; doi:10.1186/s12966-025-01795-8)
Supplement: Supplementary file 1 — Supplementary Material 1 [file 12966_2025_1795_MOESM1_ESM.docx]

| **Appendix 1. Study participant flow diagram for the Nurses’ Health Study 3 Mobile Health Substudy and restriction criteria for primary analytic dataset for cohort population (N) and GPS observations (n).** |
| --- |
| 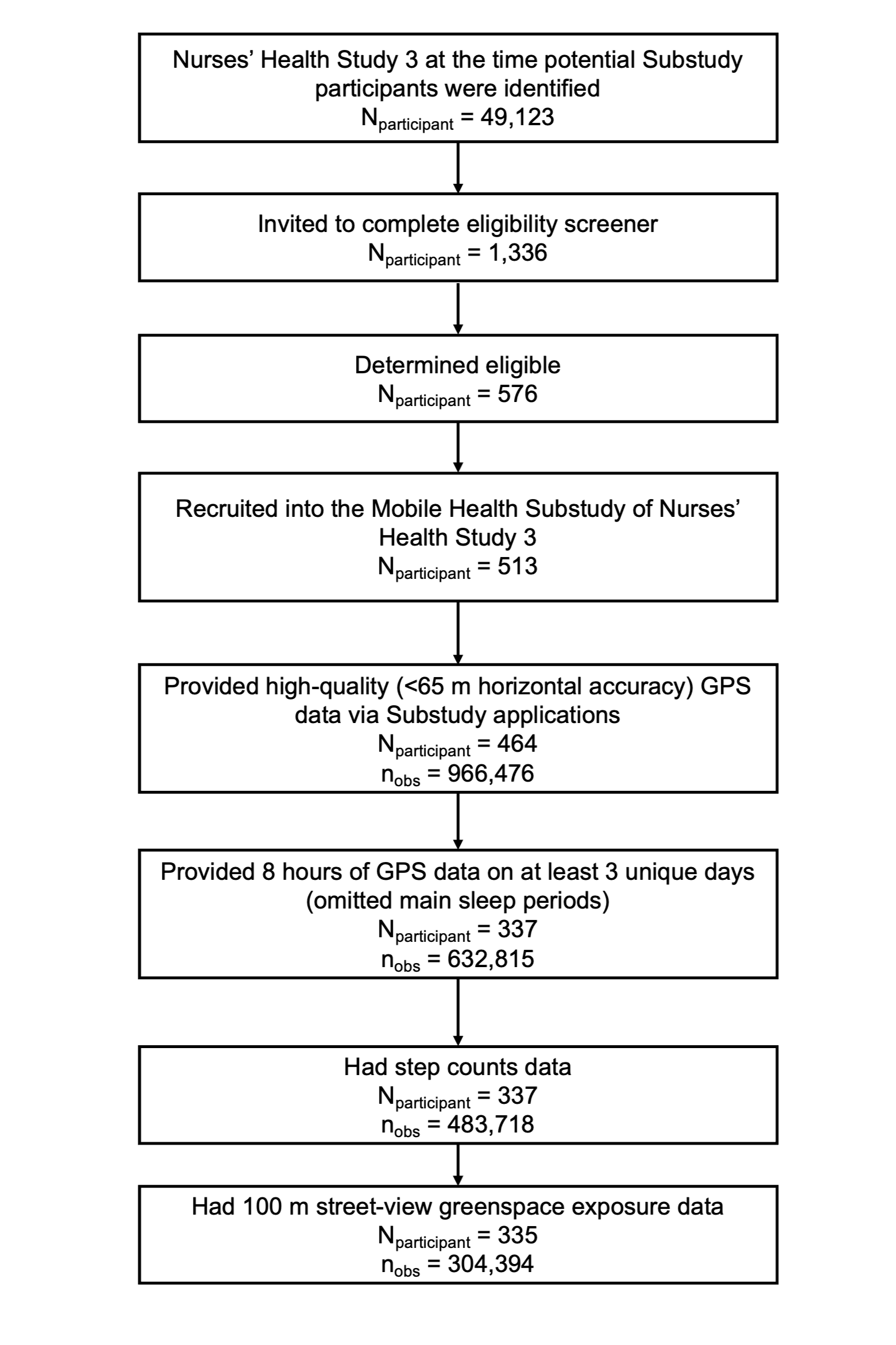 |

| **Appendix 2. Nurses’ Health Study 3 mHealth Substudy characteristics by included and excluded participants, and across restriction criteria.** | | | |
| --- | --- | --- | --- |
|  | **Excluded Participants** | **Primary Analytic Dataset**  (3 unique days with 8 hours of data) | **Secondary Analytic Dataset**  (10 unique days across minimum two study periods with 12 hours of data) |
|  | (N = 178) | (N = 335;  304,394  Observations) | (N = 206;  232,403  Observations) |
| Age, mean(SD) | 39.0 (7.1) | 39.4 (7.0) | 39.2 (6.9) |
| Race, (%) |  |  |  |
| White | 94.8 | 92.0 | 91.0 |
| Black | 0.6 | 3.2 | 3.5 |
| Asian | 1.7 | 0.7 | 0.9 |
| Mixed Race | 2.3 | 0.9 | 0.5 |
| Other | 0.6 | 3.2 | 4.2 |
| Hispanic, (%) | 5.1 | 5.7 | 5.7 |
| Married, (%) | 54.5 | 60.1 | 59.5 |
| Advanced Degree, (%) | 23.0 | 25.9 | 25.7 |
| Employment, (%) | 94.6 | 97.1 | 97.4 |
| Walkability z-score, mean(SD) |  | 0.7 (3.7) | 0.7 (3.5) |
| Neighborhood SES z-score, mean(SD) |  | 1.9 (3.3) | 1.9 (3.3) |
| Temperature (°C), mean(SD) |  | 14.8 (9.9) | 14.6 (10.0) |
| Precipitation (mm), mean(SD) |  | 3.4 (9.0) | 3.3 (9.0) |
| NDVI (0-1), mean(SD) |  | 0.3 (0.2) | 0.3 (0.2) |
| Seasonality, (%) |  |  |  |
| Fall |  | 25.9 | 26.9 |
| Spring |  | 22.0 | 20.7 |
| Summer |  | 32.8 | 31.8 |
| Winter |  | 19.3 | 20.6 |
| Abbreviations: SES, socioeconomic status; NDVI, normalized difference vegetation index. | | | |

| **Appendix 3. Participant Street-view Greenspace and Physical Activity Distributions Across Seasons in the Nurses’ Health Study 3 mHealth Substudy (N = 335, n = 304,394 10-minute observations).** | | | | | |
| --- | --- | --- | --- | --- | --- |
|  | Overall  n = 304,394 observations | Fall  n = 78,710 observations | Spring  n = 67,067 observations | Summer  n = 99,949 observations | Winter  n = 58,668 observations |
| Mean steps-per-minute, mean(SD) | 6.9 (14.6) | 6.7 (14.6) | 6.9 (14.3) | 7.1 (15.0) | 6.6 (14.4) |
| Street-view trees (%), mean(SD) | 17.3 (12.9) | 17.1 (13.0) | 17.6 (12.8) | 16.8 (12.6) | 17.9 (13.3) |
| Street-view grass (%), mean(SD) | 7.5 (6.3) | 7.4 (6.2) | 7.6 (6.4) | 7.7 (6.2) | 7.5 (6.3) |
| Street-view other greenspace (%), mean(SD) | 1.2 (1.8) | 1.2 (1.8) | 1.3 (2.0) | 1.2 (1.8) | 1.2 (1.8) |
|  |  |  |  |  |  |
|  | Overall  n = 304,394 observations | Midwest  n = 92,881 observations | Northeast  n = 49,535 observations | South  n = 91,248 observations | West  n = 70,730 observations |
| Mean steps-per-minute, mean(SD) | 6.9 (14.6) | 6.5 (14.1) | 7.3 (15.7) | 6.8 (14.2) | 7.0 (15.1) |
| Street-view trees (%), mean(SD) | 17.3 (12.9) | 18.3 (13.1) | 17.0 (13.3) | 17.3 (12.3) | 16.1 (12.9) |
| Street-view grass (%), mean(SD) | 7.5 (6.3) | 9.7 (5.8) | 7.6 (6.8) | 8.8 (6.5) | 3.1 (3.1) |
| Street-view other greenspace (%), mean(SD) | 1.2 (1.8) | 0.9 (1.4) | 1.0 (1.3) | 1.0 (1.8) | 2.0 (2.5) |

| **Appendix 4. Correlations between street-view exposures and other spatial factors in analytical data in the Nurses’ Health Study 3 mHealth Substudy (N = 335, n = 304,394 10-minute observations).** |
| --- |
| 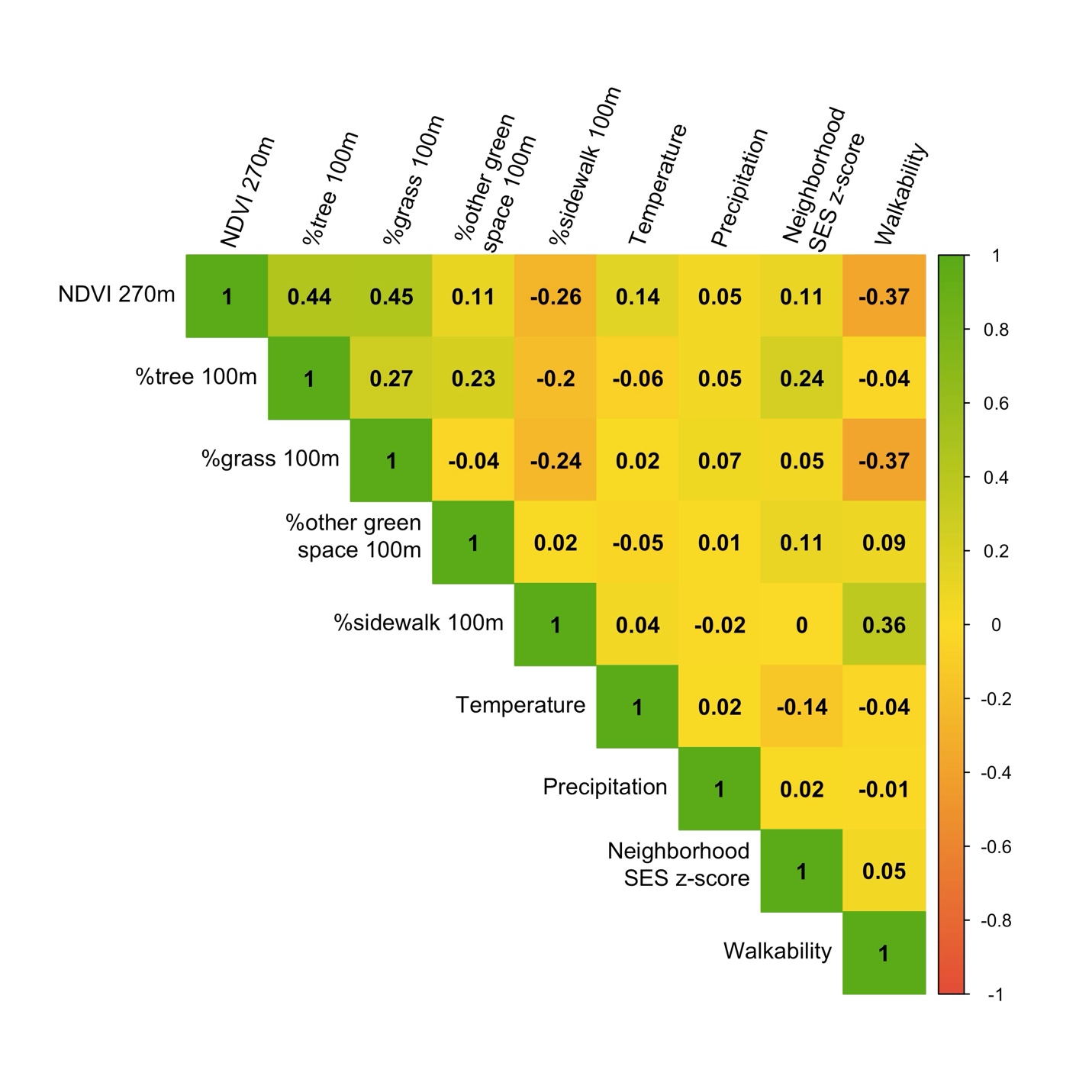 |
| *Notes.* NDVI = Normalized Difference Vegetation Index. SES = socioeconomic status. |

| **Appendix 5. Results of sensitivity analyses^a^ on associations between three street-view greenspace metrics^b^ and average steps-per-minute across a 10-min period^d^ accounting for potential biases in the Nurses’ Health Study 3 mHealth Substudy (N = 335, n = 304,394 10-minute observations).** | | |
| --- | --- | --- |
| **Variable** |  |  |
| A. Trees, % | B. Grass, % | C. Other greenspace, % |
| 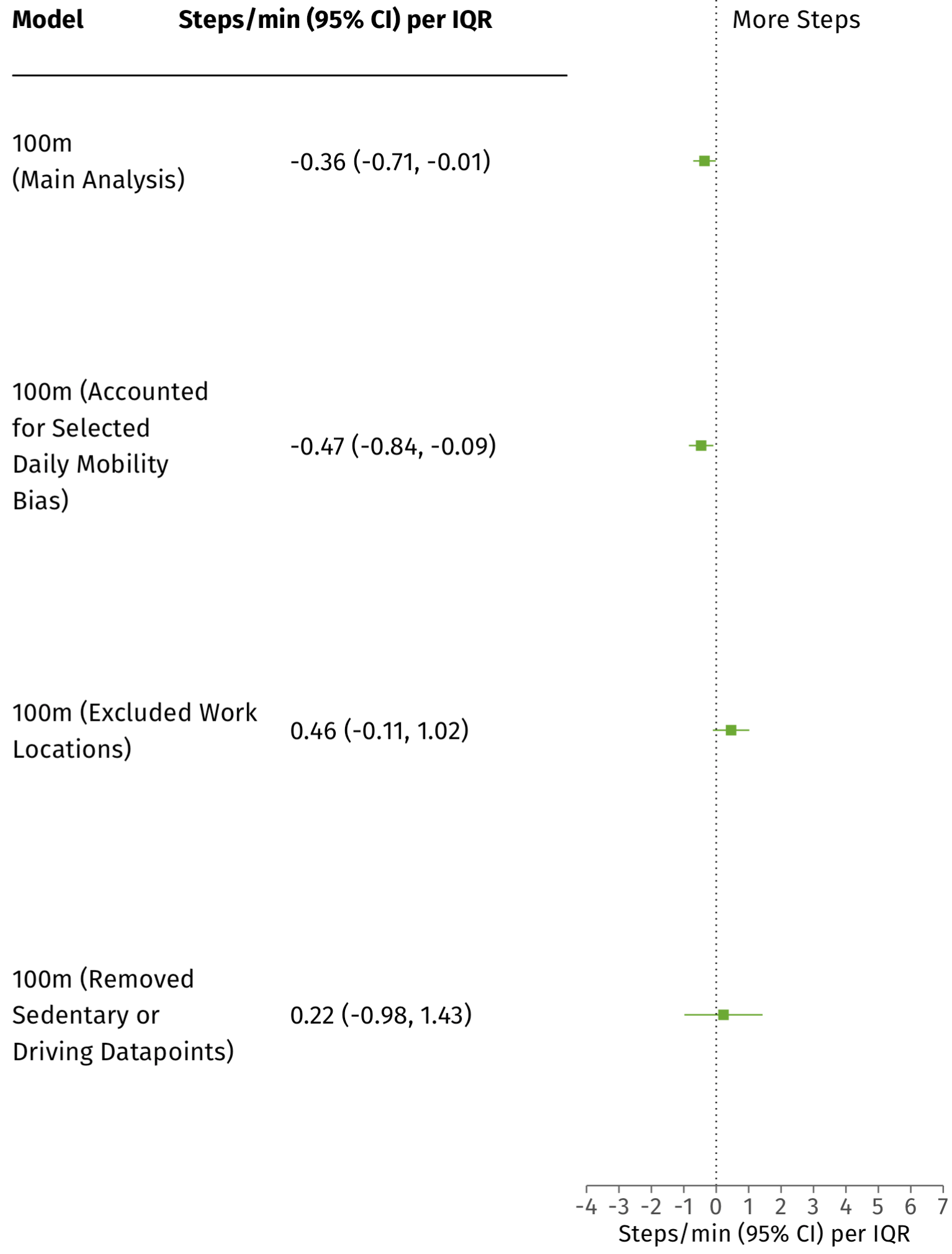 | 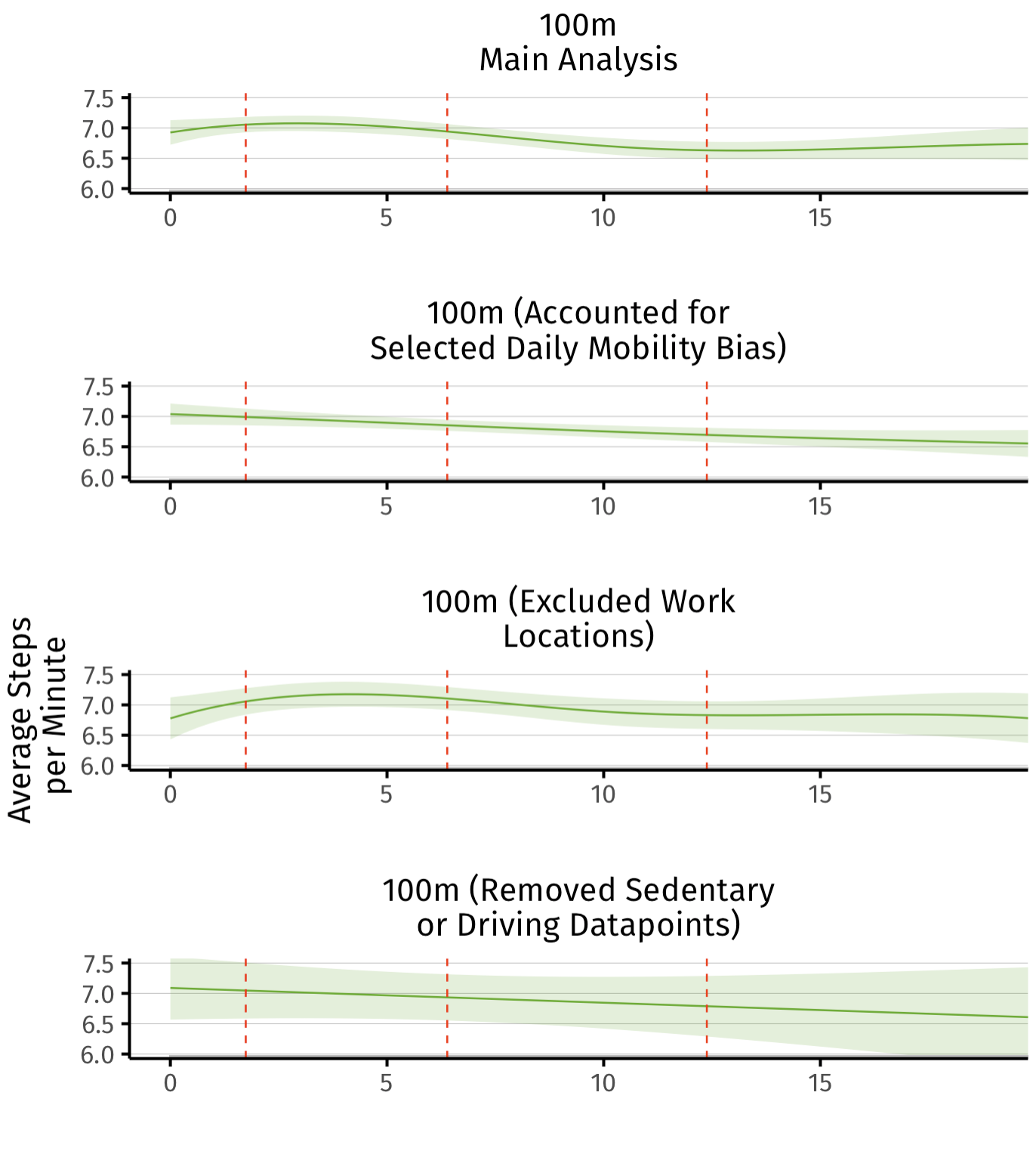 | 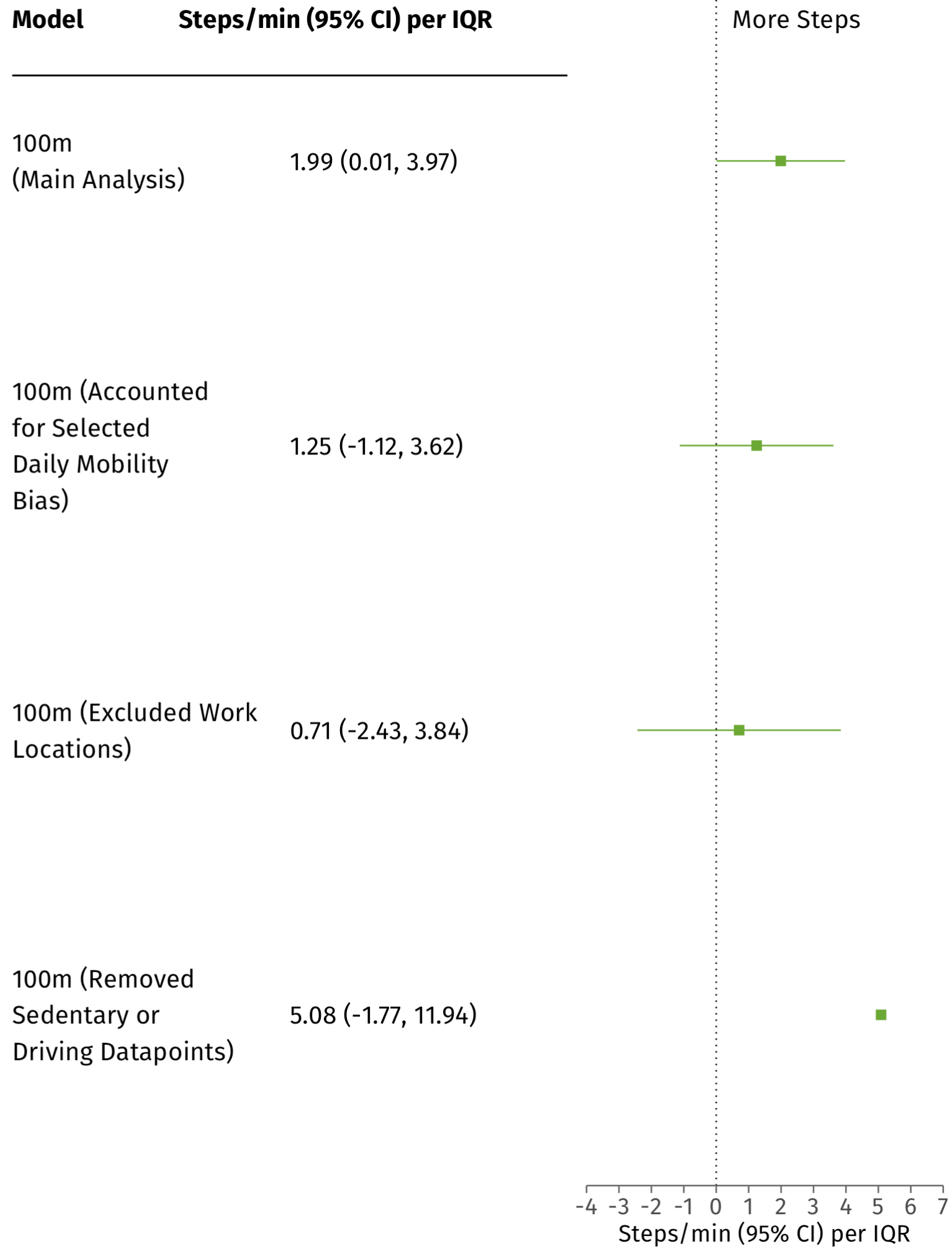 |
| ^a^Model notes.  For models accounted for selective daily mobility bias. We restricted activity space to GPS locations within a standard deviation ellipse — subject-specific standard deviation of the x-coordinates and y-coordinates from the mean center of that subject’s points, to eliminate locations outside of an individual’s normal range.  For models excluded work locations. We omitted time at work by geocoding workplace addresses at the time of study and restricting GPS location data to locations outside of a 160-m radial buffer (0.1 mile).  For models with sedentary or driving datapoints removed. We used timestamps in addition to GPS locations to estimate velocity, and restricted analyses to velocities that fell between walking and running (0.8–4 m/s) to obtain datapoints of active transport or recreating.  ^b^ Generalized Additive Mixed Models (GAMM) was applied to examine potential linear and non-linear associations between GPS-based street-view greenspace exposure and PA. We included a random intercept for each participant to account for repeated measurements within the same participant. Three street-view greenspace metrics were mutually adjusted in all models. Trees and other greenspace were modeled with linear terms; while grass was modeled with a non-linear term.  ^c^25^th^, 50^th^, and 75^th^ percentiles of each street-view metric were displayed as red dash lines, along with actual values. The x-axis was limited to 5^th^ and 95^th^ percentile of each metric to restrict influences of extreme estimates on visual.  ^d^Models controlled for age, education level, marital status, and area-level measures of neighborhood socioeconomic status, walkability, mean daily temperature, daily precipitation, season and Census region in the 2018–2020 Nurses’ Health Study mHealth Substudy. | | |
